# Speaking Truth to Power: Twitter Reactions to the Panama Papers

**DOI:** 10.1007/s10551-018-3997-9

**Published:** 2018-08-30

**Authors:** Dean Neu, Gregory Saxton, Jeffery Everett, Abu Rahaman Shiraz

**Affiliations:** 1 York University, Toronto, Canada; 2 University of Calgary, Calgary, Canada

**Keywords:** Whistleblowing, Stakeholders, Social accountability

## Abstract

The current study examines the micro-linguistic details of Twitter responses to the whistleblower-initiated publication of the Panama Papers. The leaked documents contained the micro-details of tax avoidance, tax evasion, and wealth accumulation schemes used by business elites, politicians, and government bureaucrats. The public release of the documents on April 4, 2016 resulted in a groundswell of Twitter and other social media activity throughout the world, including 161,036 Spanish-language tweets in the subsequent 5-month period. The findings illustrate that the responses were polyvocal, consisting a collection of overlapping speech genres with varied thematic topics and linguistic styles, as well as differing degrees of calls for action and varying amounts of illocutionary force. The analysis also illustrates that, while the illocutionary force of tweets is somewhat associated with the adoption of a prosaic and vernacular ethical stance as well as with demands for action, these types of voicing behaviors were not present in the majority of the tweets. These results suggest that, while social media platforms are a popular site for collective forms of voicing activities, it is less certain that these collective stakeholder voices necessarily result in forceful accountability demands that spill out of the communication medium and thus serve as an impulse for positive social change.

In helping make once-private information public, social media has furthered the aggregation and channeling of public voices. Connecting large numbers of geographically distant actors in short periods of time, it has become a key component in a *chain of activity* that seeks to hold the powerful accountable (Earl et al. 2015; Fieseler and Fleck 2013). This potential of social media has not gone unnoticed. Non-governmental organizations, most notably Wikileaks and the International Consortium of Investigative Journalists (ICIJ), are using it in an attempt to animate and facilitate grassroots accountability. International financial institutions, such as the World Bank and USAID, have started to emphasize social accountability and to utilize social media-based accountability mechanisms to help “bridge the accountability gap between citizens and governments” (O’Meally 2013; USAID 2018). Governments are also taking up social media, incorporating it into “open government initiatives” (Unsworth and Townes 2012) as well as using it to respond to criticisms. Not surprisingly, corporate actors have also embraced social media as a means of both improving stakeholder relations and responding to external threats (cf. Fieseler et al. 2010; Schulze-Horn et al. 2015; Hossain et al. 2018).

While institutional actors assume that social media can help hold powerful business and government actors accountable, little is known about how these chains of activity function in practice. For example, what are the characteristics of social media-based voicing behaviors? How are normative notions of “right and wrong” enlisted within voicing activities? Are calls to action a salient component? Do the public voices of individuals coalesce into a collective form of stakeholder voice? And, finally, when do social media participants “respond” to the voicing activities of others? It is these details, we propose, that influence if, when, and how individual public voices congeal into stakeholder voice(s) that sometimes “spills out” of communication media, thereby providing the impulse for social activism and positive social change.

To tentatively answer some of these questions, the current study examines the micro-linguistic details of Twitter responses to the whistleblower-initiated publication of the Panama Papers. The Panama Papers consist of 11.5 million documents from Mossack Fonseca, an offshore Panamanian-based law firm, which were leaked to the German newspaper *Süddeutsche Zeitung* and then shared with the International Consortium of Investigative Journalists (ICIJ) and its partners. The leaked documents contained the micro-details of tax avoidance and tax evasion schemes used by business elites. They also contained information on the politicians and government bureaucrats who had managed to accumulate significant financial wealth, either before or during their period of public service. Included in the documents, and foregrounded in the initial newspaper exposé, were two former presidents and the then-current president of Argentina, the then-current president of Ecuador, well-known business figures from Central and South America, and “local” sports hero Lionel Messi. The public release of the documents on April 4, 2016 resulted in a groundswell of Twitter and other social media activity throughout the world, including 161,036 Spanish-language tweets in the subsequent 5-month period.

The resulting social media activity allows us to consider three important aspects of voicing behaviors. First, we analyze how Twitter participants respond to the publication of previously private information regarding the wealth-accumulation and concealment activities of politicians and tax-avoidance strategies of wealthy individuals—including whether responses to these activities and strategies are individualized, polyvocal, or homogeneous. Starting from the insights of Bakhtin (2013), we examine whether the Twitter communication stream consists of multiple speech genres, each with different subject matters and linguistic styles of voicing. Second, we consider how ethical stances are enlisted within different speech genres to both make sense of the events and to evaluate the activities of the involved participants. Finally, we analyze the association between tweet characteristics and whether the tweet incities a response on the part of other Twitter participants. This response—what we refer to as the *illocutionary force* of the tweet—is important because it helps to extend and diffuse the conversation as well as to channel the conversation into something that has the potential to spill out of the communication medium. According to Butler (2015), it is this “spilling out” that is key to holding the powerful accountable and to positive social change.

The findings, not surprisingly, illustrate that there was a significant public reaction to the publication of the Panama Papers and that a large and diverse group of individuals participated in the subsequent conversations. These responses were not homogeneous; rather, they exhibit a collection of overlapping speech genres with varied thematic topics and linguistic styles, including ethical stances and call for action, as well as different amounts of illocutionary force. This finding suggests that there are a series of different collective voices and, hence, different groups of stakeholders. Second, the analysis illustrates that, while the illocutionary force of tweets is somewhat associated with the adoption of a “common sense” style of ethical speaking (what we refer to as a prosaic and vernacular ethical stance) as well as with demands for action, these types of voicing behaviors *were not* present in the majority of the tweets. These results suggest that, while social media platforms are a popular site for collective forms of voicing activities, it is less certain that these collective voices necessarily result in forceful accountability demands that spill out of the communication medium and thus serve as an impulse for positive social change.

## Social Media-Based Voices

The coming together of individuals in public spaces to voice their opinions and analyses of the events of the day has, historically, taken various forms. For example, “enlightenment” voicing relies on reasoned discussion and debate and views such discussion as a form of dialogic citizenship within the public sphere (cf., Habermas 1962). This vantage point sees the public sphere as “an essential component of sociopolitical organization because it is the space where people come together as citizens and articulate their autonomous views to influence the political institutions of society” (Castells 2008, p. 78). The public sphere, moreover, can exist in “the pages of diverse journals and periodicals,” as well as in physical public spaces (Gardiner 2004, p. 28). In addition, it is reasoned discussion and debate within the public sphere that is the starting point for advocating for social change (Gardiner 2004, p. 28; Castells 2008, p. 79). This vantage point, at least as articulated by Habermas, acknowledges the role of newspapers, etc., in providing a discussion and dissemination forum for public voices but is suspicious of the ways that privately owned media filters and shapes “public” voices (Gerhards and Schäfer 2010, p. 145).

In contrast to this first form, “carnivalesque” voicing draws its inspiration from the “medieval carnival.” This voice relies on parody and linguistic excess to unmask and challenge the powerful (Bakhtin 1994). This form of voice is a type of linguistic performance that speaks “folk truth to power.” This form of voice, it should be noted, is also often less optimistic about potential performative consequences, since social and economic systems have an infinite capacity to absorb dissent (Robinson 2011). Carnivalesque voice acts are emotive yet reasoned responses that view the act of speaking truth to power as an act of citizenship and as a “victory over fear”—even if the performative consequences do not extend beyond the exercising of one’s right to speak (Bakhtin 1994, p. 209). Like enlightenment voice, carnivalesque voice has a long lineage stretching back to at least the middle ages. While there are undoubtedly other historical forms of citizen voicing activities (cf. Spivak 1988), these two examples draw attention to the potential range and styles of voicing activities, including the possible hybridized forms that may co-exist within a public sphere.

The emergence of social media has increased the quantity of discursive spaces where voicing can occur as well as the speed by which these voices are disseminated and heard (Gerbaudo 2012; Tufekci 2017). In turn, social media-based voices have come to occupy an important role in current-day chains of activity that attempt to hold the powerful accountable (Castelló et al. 2013; Lyon and Montgomery 2013). As mentioned in the introduction, the recent initiatives of Wikileaks, the ICIJ, the World Bank, and others presume that the making-public of previously private information will animate social media-based public discussion and that this discussion will, in turn, provide the impulse for other activities that result in social change. In this regard, social media is a public arena of citizenship (Whelan et al. 2013; Butler 2015) and voicing activities are a communication event that puts into motion a series of other actions, actions that challenge, and sometimes shift existing relations of force so as to constitute a new result (Hall 1988, p. 132). In addition, social media allows ordinary citizens to speak “truth to power” in a way that not only aggregates and amplifies individual voices (Bonilla and Rosa 2015, p. 6), but also hopefully spills out of the communication medium into the surrounding world.

To help us understand the attributes and consequences of social media-based voicing activities, we rely on work rooted in the discipline of linguistics.^1^ This work suggests that tweets be viewed as a type of utterance (Bakhtin 2013, p. 71) that is placed by individuals into the Twitter communication stream. Second, this work proposes that the illocutionary force (Bauman and Briggs 1990, p. 62) of individual tweets depends, in part, on the style of voicing activities. The remainder of this section outlines the key contours of this perspective.

Tweets—as the base element and “the real unit of speech communication” (cf. Bakhtin 2013, p. 71) within Twitter—are placed into what Butler (2015, p. 72) refers to as a digital space of appearance.^2^ Digital spaces of appearance such as Twitter and Facebook act as a repository for overlapping, yet distinct, speech genres (Bakhtin 2013). Social media spaces of appearance are partially structured spaces where individuals can voice their opinions and emotions *within* the constraints of the medium (Marquez 2012, p. 29; Bonilla and Rosa 2015, p. 7). These constraints include the length of the utterance, the types of language and opinions that can be used, the algorithms that influence #hashtag trends, and how Twitter will use the information on the participants. Thus while Twitter and other social media sites may appear to be a site for “free speech,” the aforementioned constraints mediate who participates and the nature of the utterances.^3^

Individuals exercise their speech will (Bakhtin 2013) not only by using Twitter as their communication medium but also by placing their tweet into one of the pre-existing speech genres: “the speaker’s speech will is manifested primarily in the *choice of a particular speech genre*” (2013, p. 78, emphasis in original). Speech genres, according to Bakhtin, are relatively stable forms of speech communication that are characterized by somewhat unique subject matters and linguistic styles. They not only pre-date individual utterances but also are tacitly known to speaking subjects:

…we speak only in definite speech genres, that is, all our utterances have definite and relatively stable typical forms of construction of the whole. Our repertoire of oral (and written) speech genres is rich. We use them confidently and skillfully in practice… (Bakhtin 2013, p. 78)

According to Bakhtin, “speech communication would be almost impossible” (p. 79) without a knowledge and mastery of multiple speech genres. Bonilla and Rosa’s (2015) research on the Twitter #Ferguson digital protests illustrates how different speech genres co-exist within a single Twitter #hashtag.

The idea that the stream of individual tweets within a Twitter #hashtag consists of multiple speech genres is important for two reasons. First, speech genres are the mechanism by which individual voices accumulate, congeal, and are channeled into a collective stakeholder voice (cf. Bonilla and Rosa 2015, pp. 5–6; Markham 2014; Gerbaudo ands; Treré 2015). Speech genres both bring together individual voices and partially structure these voices. Communication streams such as Twitter offer potential participants a menu of pre-existing speech genres each with somewhat unique linguistic style for expressing one’s emotions and opinions. These speech genres, by inviting the participation of individuals, help to construct stakeholder groups that speak in the name of “we the people” (Butler 2015, p. 156). Butler suggests that the collective voice of the different stakeholder groups articulates declarative statements that performatively enact forms of popular sovereignty (p. 176).

Second, identifying groupings of individual voices that share a subject matter and linguistic style—i.e., a speech genre—within a #hashtag provides us with a way to identify the presence of different stakeholder groups and to better understand the differences in orientation and ethical stances across groups (cf. Ruf et al. 2001; Beekun and Badawi 2005; Neville and Menguc 2006; Parent and Deephouse 2007). It also provides a starting point for examining the illocutionary force associated with different types of tweets. Similar to corporate-style utterances that attempt to generate a type of illocutionary force via repetition and the use of techniques of persuasion (cf. Suddaby et al. 2015; Hossein et al. 2018), it is the accumulation of individual voices into a collective that, in part, determines the illocutionary force of social media reactions. Furthermore, as prior research suggests, collective stakeholder voices are arguably a precondition for getting the attention of the powerful and for speaking truth to power in a way that requires the powerful to respond (cf. Tufekci 2017). While governments and businesses have a normative responsibility to recognize different stakeholder groups (Freeman 1994, 2010), it is the illocutionary force of voicing activities that insists that certain stakeholder groups be taken into account: that demands accountability (Bonilla and Rosa 2015, p. 7). From this vantage point, stakeholders become relevant when their voices signal their potential to act against the powerful (cf. Oliver 1991). This potential is conveyed, in part, by the illocutionary force of collective stakeholder voices that makes these normative responsibilities into something that the powerful must “respond to” and “act on.”

The provided linguistic perspective accepts that social media-based public voices can act as an important linchpin in chains of activity that seek to hold the powerful accountable; however, it emphasizes that such outcomes depend in part on the composition and linguistic attributes of the communication stream. Twitter responses to events such as the publication of the once-private Panama Papers may consist of multiple speech genres (including the aforementioned enlightenment and carnivalesque styles of voicing); furthermore, these different voicing behaviors can have different linguistic styles and differing amounts of illocutionary force. For these reasons, it is important to examine the attributes of stakeholder voices in detail.

**Fig. 1:**
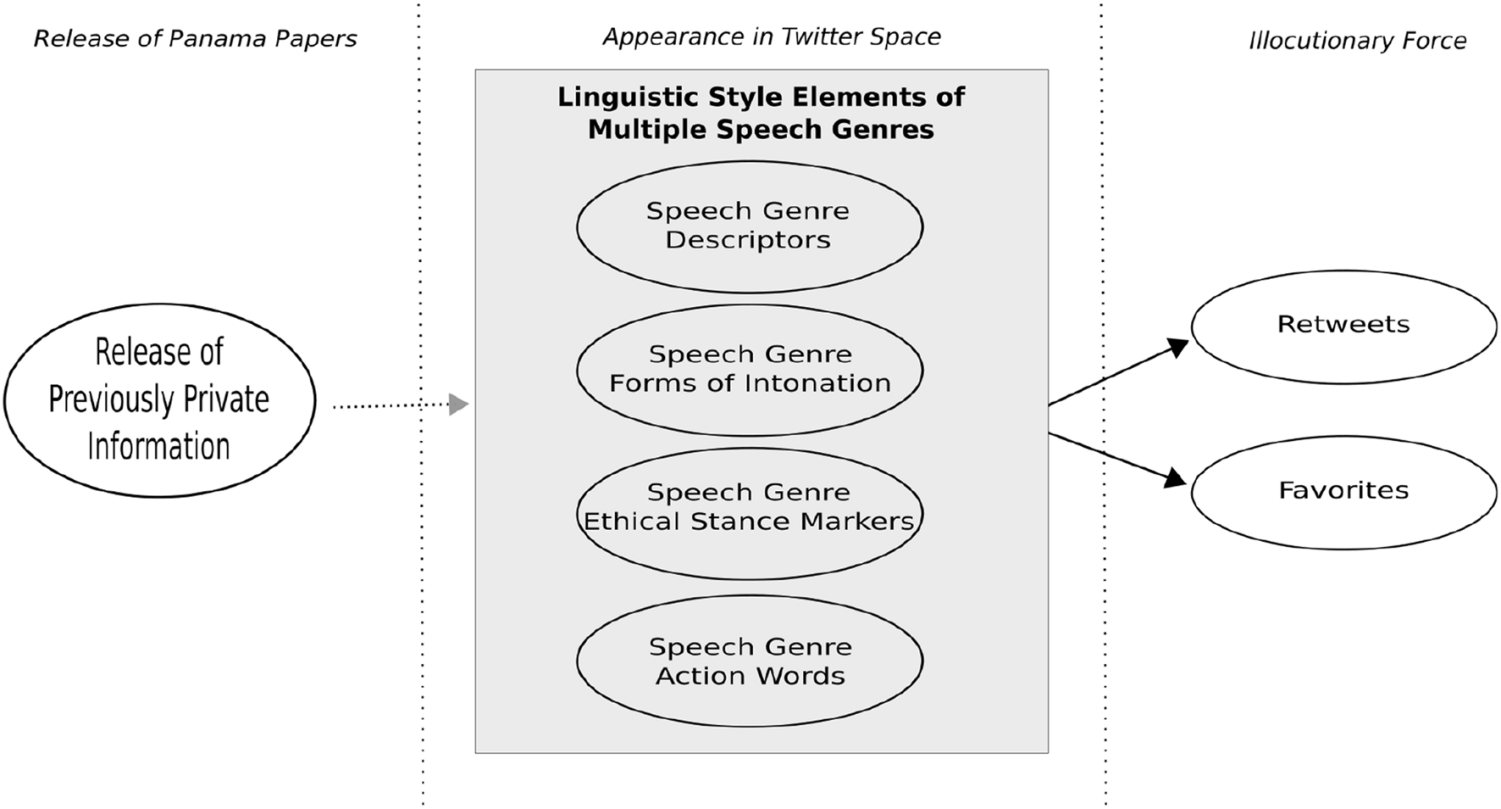
Connections among core theoretical concepts. Empirical tests focus on the middle and right blocks: the speech genres, the linguistic style elements, and the measures of illocutionary force

Figure 1 provides a diagram of the connections among these core theoretical concepts—the publication of previously private information, the appearance on Twitter of multiple speech genres with different linguistic styles, and illocutionary force. As elaborated in the following section, our empirical tests focus on the middle and right conceptual blocks, where we elaborate measures of three different speech genres, four elements of linguistic style, and two measures of illocutionary force.

## Method

**Fig. 2:**
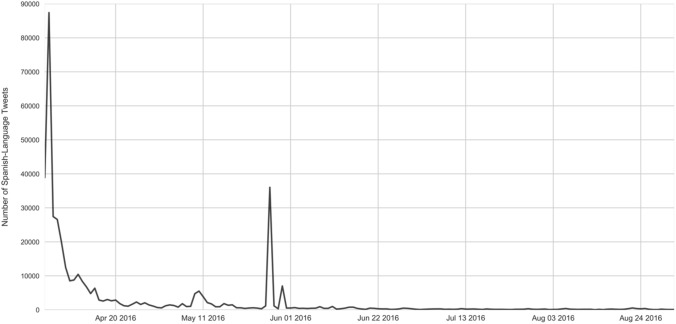
Overall Tweet volume, Spanish-language #PanamaPapers Tweets, 4/3/2016–9/1/2016

Between the date of the release of the Panama Papers (April 3, 2016) and September 1, 2016, there were 161,036 Spanish-language tweets that contained location data on the country of origin for the speaker with the hashtags #Panamagate, #Panamapapers, or #PanamaLeaks. Figure 2 shows the daily variation in tweeting frequency over the study period. This group of tweets was downloaded in real-time using a custom Python script and “cleaned” using Python and Textcrawler before being analyzed using text processing and analysis packages available in *R* (Feinerer et al. 2008; Roberts et al. 2018).

After downloading the tweets, we used a combination of *a priori* and inductive methods to tentatively identify different speech genres within the Twitter stream. Our review of the Panama Paper archive and the initial ICIJ articles (ICIJ 2018) indicated that *POLITICIANS* and *TAXES* were two potential speech genres: they point to the use of secretive offshore financial accounts by politicians, corporations, and the wealthy to hide their funds. A third tentative speech genre—the *FACTS* speech genre—was also present within the archive and initial ICIJ articles with this theme focused on describing (rather than interpreting) what the Panama Papers were about. Using our preliminary review of the ICIJ documents as background, we then examined the term-document matrix (tdm) for the corpus of 161,036 tweets. The text processing algorithm in R generates a listing of all terms that appear within the documents in the corpus. The current corpus, for example, contained approximately 9000 terms after removing infrequent terms (those with less than 250 occurrences) and stop words (i.e., “and, ” “or,” “the” etc.). We rank-ordered the terms from more to less frequent and then examined the list. The rank-ordered listing allowed us to identify the names of notable persons as well as prevalent words. The names of Latino politicians (Macri, Kirchner, Grindetti, Correa)^4^ were used to construct the *POLITICIANS* speech genre and variations of the word *TAXES* (“tax,” “taxes,” “taxation,” etc.) were used to construct the second speech genre. The *FACTS* speech genre was formed by combining the other most prevalent words and word stems (offshore, investiga, paraiso, fiscal, empresa, mossack)^5^ from this listing (excluding the variations of “Panama”).

We then used these genre-specific word lists to create tweet-level measures for the three genres. If a tweet contained a word in the *POLITICIANS* list, it is given a score of 1 on our variable *POLITICIANS*, otherwise 0; similarly for *TAXES* and *FACTS*. In other words, the three variables *POLITICIANS, TAXES*, and *FACTS* indicate whether a tweet contains words in the *POLITICIANS, TAXES*, and *FACTS* genres, respectively. Table 1 summarizes the descriptive statistics for these and all other variables used in our empirical tests. As shown in Table 1, mentions of *POLITICIANS* occurred in 15% of the tweets, *TAXES* in 2% of the tweets, and *FACTS* in 15% of the tweets. It should be noted that the conceptual appropriateness of these groupings is an empirical question: the finding of differences in linguistic style across the groupings will be a partial confirmation of the existence of distinct speech genres.

**Table 1:** Descriptive statistics

Statistic	*N*	Mean	SD	Min	Max
Speech genres					
Politicians	161,036	0.146	0.354	0	1
Taxes	161,036	0.017	0.129	0	1
Facts	161,036	0.148	0.356	0	1
Linguistic style—descriptors					
Corrupt	161,036	0.011	0.105	0	1
Evader	161,036	0.005	0.067	0	1
Thief	161,036	0.002	0.046	0	1
Fraud	161,036	0.005	0.069	0	1
Linguistic style—forms of intonation					
Exclaim	161,036	0.088	0.284	0	1
Excrement	161,036	0.002	0.05	0	1
Shame	161,036	0.006	0.074	0	1
Linguistic style—ethical stance markers					
Ethical	161,036	0.003	0.054	0	1
Moral	161,036	0.006	0.075	0	1
Transparent	161,036	0.005	0.072	0	1
Rich	161,036	0.006	0.077	0	1
Poor	161,036	0.004	0.061	0	1
Linguistic style—action words					
Should	161,036	0.019	0.138	0	1
Demand	161,036	0.002	0.044	0	1
Illocutionary force					
Retweeted	161,036	3.796	25.525	0	3,158
Favorited	161,036	2.294	17.506	0	2,753

As mentioned in the theoretical framing above, we expect that the different speech genres will contain different linguistic styles. Consequently, we consider four elements of linguistic style: descriptors, forms of intonation, ethical stance markers, and calls to action. The linguistic markers that we use are based on our knowledge of the speaking context within which the tweets were created (cf. Bakhtin 2013, pp. 90–91). Our social location in the Latino world and fluency in Spanish provided us with the necessary contextual knowledge to identity relevant linguistic markers. As with the three speech genre variables, for all of the following linguistic style variables, we use indicator (0,1) variables because they are easier for the reader to interpret (the results did not change when we used continuous measures versus indicator variables). Each variable described below thus indicates whether a tweet contains one or more of the relevant words/linguistic tokens (see Table 1 for summary statistics).

*Descriptors* are words that “point to” and index (Peirce 1932; Silverstein 1976, p. 48; Nakassis 2013, p. 56) as well as key (Goffman 1974) underlying frames of meaning and sense-making. The descriptor words *corrupt* (corrupto), *evader* (evasor), *thief* (ladron), and *fraud* (fraude) are linguistic tokens (cf., Derrida 1977) that point to an abstract type of imaginary figure (what Derrida refers to as a linguistic type). All four of these words are primarily used as nouns to describe the actions of the individuals named in the Panama Papers. Such descriptors “describe,” albeit in ways that are somewhat evaluative (cf., Kockelman 2004, p. 144).

*Intonation* refers to the use of expressive punctuation and words of linguistic excess. These linguistic signs foreground the expressive aspects of communication (Jakobson et al. 1951, p. 15; Christie 2013) and operate as metacommunication about communication (Ruesch and Bateson 1968, p. 209). Exclamation marks (*exclaim*) within written utterances are a visible emotive practice similar to raising one’s voice in verbal conversation. The use of the words *excrement* (mierda) and *shame* (averguenza), for example, are common forms of expressive intonation in Spanish (Fernández 2006). Phrases such as “qué mierda” (what sh*t) and “él es una mierda” (he is a sh*t) are used to express the emotive and evaluative opinion of speakers. Likewise, “qué vergüenza” (shameful or shame on him/her) provide a more polite way of expressing the same opinion. The use of words of linguistic excess is characteristic of carnivalesque forms of voicing where abusive language, insulting words, and references to bodily functions are used to mock the powerful (Bakhtin 1994, pp. 203–204).

*Ethical stance* is identified by words such as *ethics, morals*, and *transparency*, as well as localized tokens such as the *rich* and the *poor*. These markers simultaneously key that the speech topic both involves normative questions of right and wrong (Briggs and Bauman 1992, p. 144) and invokes pre-existing value systems (Keane 2011, p. 174). Such linguistic signs operate as stance markers that signal the “evaluative and intentional commitment that speakers take towards states of affairs” (Kockelman 2004, p. 127). Words such as “ethics” directly signal that this is a speech event “about” ethics and thus brings the events or people into a relationship with these terms. This relationship can be expressed in a declarative statement such as “s/he is unethical,” or by asserting that “ethics etc. are important.” While references to ethics can be viewed as a form of enlightenment reasoning and voicing, the use of vernaculars that draw attention to the inequalities inherent in the existing class structure also point to questions of right and wrong (Bakhtin 1994, p. 209). Within the Latino world, the words *pobres* (the poor) and *ricos* (the rich) are often used to both locate an utterance vis-à-vis a class structure and to signal what the speaking subject thinks or feels is relevant.

*Call to action* words (*should* and *demand)* are directed at other Twitter participants. Prior research suggests that calls to action within communication streams are important because it both keys that something should be done and explicitly indicate a path of action (Gerbaudo 2016, p. 55). Calls to action direct participants to do something thereby connecting the world of linguistic signs and the world of concrete actions: signaling how utterances can and should spill out of the communication stream into the material world (Bennett and Segerberg 2012). For these reasons, we expect that calls to action will vary across speech genres and will be positively associated with the illocutionary force of individual tweets.

To measure illocutionary force, we use *FAVORITED* and *RETWEETS* as measures. Previously, we proposed that the illocutionary force of a tweet can be characterized, in part, by the reaction to the tweet. Utterances presume an “answer” (Bakhtin 2013, p. 94) and thus more forceful tweets are those that incite a response.

Favoriting (liking) a tweet is an expressive, emotive, or cognitive response that signals that the tweeted message resonates with the tweet recipient (Alhabash and McAlister 2015). Similarly, retweeting signals that the tweet had value for the recipient and is worthy of being circulated through one’s social network. Both types of linguistic response contribute to the illocutionary force of the initial tweet and to the aggregate illocutionary force of the speech genre.

Although not reported in Table 1, the subsequent analyses also include control variables for each of the countries from which a tweet originates (i.e., a country fixed effects model).

## Results: Stakeholder Voices

The logit regression results presented in Table 2 are consistent with our proposal that the Twitter stream consists of at least three distinct forms of stakeholder voice. The *POLITICIANS* genre appears to be a carnivalesque style of voicing that uses the descriptors *corrupt* and *evader* as well as *excrement* as a form of intonation. There were numerous tweets talking about Argentina’s President and his use of offshore accounts. Comments such as “the sh*t returns to you President Macri. The lies that you told return and slap you in the head” and “all of the Argentinian politicians are sh*t and corrupt and all are the same. How sad.” While the genre is very expressive, it did not enlist ethical referents: indeed, four of the five possible ethical stance markers (*rich, moral, ethical, transparent*) were negatively associated with the genre. The *FACTS* genre, in contrast, is a type of voice that is akin to a media-based style of reporting where there is some use of descriptors (*evader* and *fraud*) but intonation and ethical referents are not as likely to be enlisted. The *TAXES* genre seems to be a hybrid between enlightenment and carnivalesque voicing styles in that there is some intonation (*excrement* is used) but there is also an explicit ethical stance in that the notions of the *poor, rich*, and *moral* are enlisted. Examples from this genre include “the rich and powerful hide their riches and evade taxes,” “the system doesn’t function, the rich evade and the poor pay,” and “paying taxes is for the poor”.

**Table 2:** Citizen voices—logit regressions with three speech genres as dependent variables

	Politicians	Taxes	Facts
Descriptors			
Corrupt	0.500***	0.007	− 0.714***
	(0.06)	(0.19)	(0.09)
Evader	0.206**	2.276***	0.231**
	(0.10)	(0.11)	(0.10)
Thief	− 0.214	0.111	− 0.800***
	(0.17)	(0.38)	(0.22)
Fraud	− 0.968***	0.389*	1.267***
	(0.21)	(0.21)	(0.08)
Forms of intonation			
Excrement	0.373***	1.883***	− 1.245***
	(0.14)	(0.18)	(0.24)
Exclaim	− 0.067***	− 0.034	− 0.163***
	(0.03)	(0.07)	(0.03)
Shame	0.064	− 0.347	− 0.900***
	(0.09)	(0.32)	(0.14)
Ethical stance markers			
Ethical	− 0.872***	− 0.302	− 0.612***
	(0.23)	(0.39)	(0.17)
Moral	− 0.620***	0.557***	− 0.693***
	(0.12)	(0.20)	(0.13)
Transparent	− 0.500***	− 1.701***	− 0.360***
	(0.13)	(0.58)	(0.11)
Rich	− 1.366***	2.729***	− 0.190**
	(0.21)	(0.09)	(0.10)
Poor	0.089	1.366***	− 0.18
	(0.12)	(0.16)	(0.13)
Action words			
Should	0.259***	0.422***	− 0.349***
	(0.05)	(0.12)	(0.06)
Demand	− 0.457**	− 0.174	− 0.985***
	(0.21)	(0.47)	(0.24)
Days after	0.003***	− 0.004***	− 0.001***
	(0.00)	(0.00)	(0.00)
Constant	− 2.806***	− 4.268***	− 1.678***
	(0.039)	(0.079)	(0.026)
Country fixed effects	YES	YES	YES
N	161,036	161,036	161,036
Pseudo *R*^2^ (McFadden)	0.20	0.07	0.015
Log likelihood	− 53,567.77	− 12,865.00	− 66,655.76
*χ* ^2^	27,012***	2,059.3***	1,984***

Table shows logit coefficients with standard errors in parentheses

*p < 0.1; **p< 0.05; ***p < 0.01

The results foreground three aspects of public response. First, the communication stream is polyvocal in that each type of stakeholder voice has a different way of responding to the publication of the once-private financial information on the wealth accumulation and concealment activities of the powerful. The presence of the populist *POLITICIANS* genre is consistent with our preconceptions in that Twitter is not only a popular communication stream but also a communication stream that encourages brevity rather than longer, and more elaborate, forms of argumentation. The *FACTS* genre is consistent with suggestions that social media communication media supplement and sometimes replace traditional print and digital news reporting (Kaye and Johnson 2003; Gerhards and Schäfer 2010). While this form of stakeholder voice does not emote, adopt an ethical stance, or call for action it does circulate and re-circulate factual information within the Twitter communication stream. The *TAXES* genre, in contrast to the other two genres, does take an explicit ethical stance. Taken together, the results illustrate that stakeholder voicing behaviors are not homogeneous but rather consist of unique mixtures of descriptors, intonation, ethical referents, and calls to action. This polyvocality within the Twitter stream is similar to the public sphere of early modern times where there was “a tumultuous intermingling of …. widely divergent styles and idioms of language, ranging from the serious to the ironic and the playful” (Gardiner 2004, p. 38).

Second, the results draw attention to the enlistment and positioning of ethical referents within the different forms of voice. Traditional forms of ethical reasoning—those that specifically enlist words such as *ethics, morals* and *transparency*—were mostly irrelevant within the communication stream. Furthermore, *TAXES* was the only genre that enlisted ethical referents: using the term *morals* as well as the notions of the *poor* and the *rich*. These latter two words are vernacular and prosaic linguistic signs (cf., Wall and Thomson 1993, pp. 58–59) that are grounded in the sociality of public conversations (Gardiner 1996, p. 134) and that both bring notions of right and wrong into the communication stream as well as signal the ethical stance of the speaker. This form of voicing does take a stance and does speak truth to power, albeit in ways that were “from the street” and “by the people.” That being said, and what we find especially interesting, both the *POLITICIANS* and *TAXES* genres are arguably populist and “from the street,” yet the two genres differ in not only linguistic styles but also in whether ethical referents are utilized.

Third, stakeholder voices about *POLITICIANS* and *TAXES* contain “calls to action” (cf., Bakhtin 1994, p. 220). These calls used *should* words but did not use *demand* words. In contrast, the *FACTS* genre did not use *should* or *demand* words. This is consistent with traditional forms of news reporting—and with the genre’s avoidance of ethical stances—but it results in a style of voice that does not, on the surface, complement the other two forms of voice. That being said, it is uncertain whether factual voicing activities are important in holding the powerful accountable—since factual voicing circulates and reminds participants of the facts’—or whether such utterances act as deadweight. While this genre is not a counter-voice that subverts stakeholder voices about *POLITICIANS* and *TAXES*, it arguably dilutes the visibility and perhaps the force of the other tweets. The next section considers the topic of illocutionary force in more detail.

## Results: Illocutionary Force

As mentioned in the theoretical framing, chains of action that attempt to hold the powerful accountable depend, in part, on inciting a reaction from Twitter audiences and on having these reactions spill out of the communication stream into the street. While an examination of the spilling-out consequences is beyond the current study’s scope, it is possible to consider whether certain speech genres and forms of linguistic styles within the genres are more likely to encourage reactions from other participants. Table 3 reports the results of OLS regressions using two measures of illocutionary force (*RETWEETS* and *FAVORITED*) as dependent variables. Because we are also interested in the interplay of speech genres with the linguistic descriptors, the table also includes the first-order interaction terms between the speech genres and the linguistic style components that were significant.^6^

**Table 3:** Illocutionary force—# of public reactions (*Retweeted* and *Favorited*) as dependent variables

	Retweeted	Favorited
Speech genres with interactions		
Politicians	1.862*** (0.206)	1.159*** (0.142)
Politicians × exclaim	− 2.361*** (0.595)	− 1.732*** (0.409)
Taxes	0.658 (0.536)	0.260 (0.368)
Taxes × rich	6.035*** (2.010)	3.208** (1.380)
Taxes × exclaim	− 0.672 (1.819)	− 0.514 (1.249)
Facts	1.308*** (0.188)	0.222* (0.129)
Facts × fraud	1.850 (1.907)	2.662** (1.309)
Facts × exclaim	− 1.990*** (0.675)	− 1.092** (0.463)
Descriptors		
Corrupt	− 0.644 (0.607)	− 0.611 (0.417)
Evader	1.082 (0.946)	0.595 (0.650)
Thief	− 1.125 (1.387)	− 0.694 (0.952)
Fraud	0.985 (1.159)	0.559 (0.796)
Forms of intonation		
Exclaim	− 0.772*** (0.261)	− 0.404** (0.179)
Excrement	− 2.196* (1.287)	− 1.137 (0.884)
Shame	− 0.743 (0.857)	− 0.557 (0.588)
Ethical stance markers		
Ethical	− 0.527 (1.185)	− 0.319 (0.814)
Moral	− 1.549* (0.843)	− 0.851 (0.579)
Transparent	− 0.199 (0.889)	− 0.069 (0.610)
Rich	4.180*** (0.939)	1.490** (0.645)
Poor	1.853* (1.050)	0.983 (0.721)
Action words		
Should	− 0.592 (0.460)	0.538* (0.316)
Demand	4.303*** (1.455)	2.324** (0.999)
Controls		
Number of Twitter friends	0.0001*** (0.00000)	0.00003*** (0.00000)
Days after	− 0.039*** (0.002)	− 0.026*** (0.002)
Constant	2.725*** (0.240)	1.443*** (0.165)
Country fixed effects	YES	YES
*N*	161,036	161,036
Adjusted *R*^2^	0.009	0.007
*F* statistic (*df* = 88; 160,947)	17.983***	14.386***

Table shows OLS regression coefficients with standard errors in parentheses

*p < 0.1; **p < 0.05; ***p < 0.01

The results illustrate that there are differences in the illocutionary force of the speech genres. Tweets about *POLITICIANS* tend to be retweeted and favorited more frequently as long as the tweet does not contain expressive punctuation (*Politicians* × *Exclaim*). Utterances about *TAXES* only have illocutionary force when they also mention the rich (*Taxes* × *Rich*). And tweets about *FACTS* are generally more likely to be retweeted and favorited, so long as they don’t contain expressive punctuation (*Facts* × *Exclaim*). These results highlight that tweets about certain topics are more likely to elicit reactions if the topic is accompanied by a certain linguistic style.

Surprisingly, the use of expressive punctuation (*Exclaim, Politicians* × *Exclaim, Facts* × *Exclaim*) tended to undermine the illocutionary force of the tweets. While expressive punctuation may be the vernacular of the street and a carnivalesque style of voicing, these forms of enunciation did not incite other participants to take concrete action in response to the tweet. The volume of one’s voice—in this case the equivalent of shouting in a verbal conversation—meta-communicates to the audience that the message animator is agitated, but it isn’t more likely to incite an audience response (Goffman 1974, p. 349). This could be because social media audiences are desensitized to these forms of metacommunication, because the metacommunication doesn’t fit the situation, or because social media audiences are responsive to the communication content rather than metacommunication (cf. Goffman 1974, pp. 352–355).

While audiences did not respond to intonation, tweets mentioning real people (*POLITICIANS*) or generic rich people (*Rich, Taxes* × *Rich*) did have illocutionary force. These linguistic tokens key figurative and real types of people, albeit people who have already been re-presented in language (Derrida 1977). As Derrida notes, tokens and types are linguistic signs that are already one step removed from real people in their materiality (p. 6). Figures of the politician or the rich *qua* linguistic types are a form of communal memory regarding how such figures are assumed to act (cf. Goffman 1974, pp. 524–526). In turn, tokens that invoke these figures are forms of popular expression regarding such “types of people.” The problem, as Butler notes, is that the sheer number of linguistic repetitions tends to naturalize these forms of self-interested (as opposed to public interested) behaviors (cf., Butler 1990, p. 146). Thus, while enunciations about politicians and the rich resonate with other participants, these forms of stakeholder voicing are ambivalent because they simultaneously reinforce and parody the initial type, thereby “breaking with” (Derrida 1977, p. 12) yet re-inscribing the type in ways that are consistent with the relations of force that hold it in place (Butler 1990, p. 147). The danger of these tweets is that they become incapable of spilling out of the communication stream because they reinforce the perception that this is how things have always been rather than prefiguring what might be (Srnicek and Williams 2015, pp. 34–35). Stated differently, this form of stakeholder voice always runs the risk of becoming ritualistic behavior that is performed for self and for other but without any expectation that things will change (Heller 2017).

On a more positive note, *demands* for action also tended to be retweeted and favorited. This finding is consistent with the premise that social media-implicated chains of activity can help to incite linguistic actions that attempt to hold the powerful accountable. The problem, of course, is that only 305 tweets out of 161,000 *demanded* action. While demands for action had illocutionary force, these tweets were drowned out by the sheer number of tweets that did not use *should* or *demand* words.

## Discussion

This study examines how individuals respond, via Twitter, to the publication of once-private information regarding the wealth accumulation and concealment activities of politicians and business people. Starting from the premise that these speech acts are forms of public voicing activities aimed at holding the powerful accountable, we considered the linguistic characteristics of the responses as well as the illocutionary force of different voicing styles. The results illustrate that voice is polyvocal in that the Twitter stream consists of multiple stakeholder voices—each with a mostly unique combination of descriptors, expressive punctuation, words of linguistic excess, ethical stance markers, and calls to action. These different linguistic elements were associated with differing amounts of illocutionary force.

The study contributes to our understanding of social accountability processes, including the possibility and limitations of voicing activities. First, the results show that individuals respond to the publication of previously private information on the wealth accumulation and concealment activities of the powerful in multiple ways. These responses were not homogeneous in terms of the thematic content nor linguistic style but they were organized in that the individual tweets had the appearance of being “placed” into different speech genres (Bakhtin 2013). These placements resulted in at least three stakeholder groupings that were thematically and linguistically consistent. In this regard, Twitter helped to aggregate and organize individual voices in ways that contributed to the emergence of stakeholder groupings that spoke, in varying ways, in the name of “we the people” (Butler 2015, p. 156; Tufekci 2017). We might go further and suggest that, in today’s world, social media-based spaces of appearance might be a precondition for speaking and assembling in the name of “we the people.”

Second, the analysis highlights the language of ethical voices (cf. Winkler 2011; Lucas and Fyke 2014; Blanc et al. 2017) within the tweets. Tweets did not enlist enlightenment-style ethical stance markers as evidenced by the absence of the words ethics/morals/transparency within the tweets. Rather, within the *TAXES* genre at least, words such as the rich and the poor were used to “point to” questions of right and wrong. These stance markers are prosaic and vernacular forms of ethical speech that keyed class-based hierarchical frames of meaning and did, in this way, judge the wealth accumulation and concealment activities of the powerful. As Bakhtin (1994) notes, the eschewing of abstract notions should not be interpreted as the absence of ethical deliberation; rather, such deliberations should be viewed as being embedded in the activities and realities of everyday life. Within this particular social media setting, ethical discourse was prosaic and vernacular rather than from a philosophy textbook.

While the study illustrates both the emergence of collective voices and the forms of ethical discourse that occurred within the different speech genres, the results also raised questions about whether these acts of speaking truth to power can actually make a difference. As mentioned in the introduction, a key premise of organizations such as Wikileaks and the ICIJ is that social media-based platforms will accumulate and channel public voices in ways that result in an illocutionary force that spills out of the medium and impacts the world. The analysis illustrates that tweets referencing the rich and demanding action had the most illocutionary force. Unfortunately, tweets containing demands for action were a very small percentage of the tweets. And while references to the rich were more prevalent, it is uncertain whether the use of this form of ethical stance marker simply reinforces a frame that emphasizes that the rich always win and the poor always pay. The constant repetition of this form of framing (Srnicek and Williams 2015, pp. 34–35) is ambivalent in that it has the potential to channel moral outrage as well as to reinforce the “order of things.” As Srnicek and Williams argue, stakeholder voices need to articulate pre-figurative framings that break with the past and positively imagine the future.

The provided analysis and results are a starting point for understanding how discursive spaces of appearance such as Twitter can potentially lead to collective stakeholder voices and other actions that forcefully and effectively demand social change. At the same time, more research is needed to better understand these processes. For example, we examined the Spanish-language tweeting responses to the publication of the previously-private Panama Papers. We made this research choice because the wealth accumulation and concealment activities of Latin American political and business elites were prominently featured within the released documents and because of the need to narrow the volume of the available tweet data. Additional research that examines the styles of voicing activities across languages and events (e.g., the subsequent release of the Paradise Papers in 2017) holds the potential to increase our understanding of these processes.

Additionally, the study acknowledged—but did not formally analyze—the role of Twitter in organizing citizen voices. Twitter, like other privately owned social media platforms influence who speaks, how they speak, the illocutionary force of different speech acts, *and* how the “digital traces” of these speech acts are subsequently used. Twitter facilitates a discursive space of appearance where individuals can speak truth to power and thus helps to construct and organize stakeholder groupings; however, it simultaneously creates a space of surveillance where subversive voices can be “answered” and where the involved individuals can be identified. As the recent Cambridge Analytica case involving Facebook data highlights, seemingly anonymous Internet data can be used for non-publicly-interested purposes. For these reasons, further research on the micro ways that different social media sites facilitate voicing *and* surveillance activities is needed in order to better understand the possibility and limitations of social media.

Finally, social media-based voicing is only one step in the process of effectively demanding social change. The provided results illustrate that individual voices can accumulate into collective voices and that the style of voicing impacts on the illocutionary force of the tweet. This said, we still do not understand if, when and how collective demands for change spill out of discursive spaces of appearance and impact on the “real” world. We do know that the Twitter reaction to the publication of the Panama Papers probably contributed to the resignation of Iceland’s Prime Minister (Obermayer and Obermaier 2016, p. 34). We also know that some taxation authorities opened investigations into the wealth accumulation activities of their residents in response to the publication of the Panama Papers *and* the subsequent social media furor (Dubinsky 2018). More research on these “successful” instances as well as settings where social media responses failed to dent the status quo are needed. As Tufekci (2017), Butler (2015) and others note, social media voicing is an important but fragile step in inciting forceful demands for social accountability and change.

## Footnotes

1 The language aspects of communication have also been studied in the sustainability (cf. Cho et al. 2010; Rodrigue et al. 2015) and business ethics literatures (cf. Winkler 2011; Lucas and Fyke 2014; Blanc et al. 2017).

2 Butler borrows the notion of a space of appearance from Arendt (1958).

3 Recent events involving Facebook have drawn attention to the ways that commercial interests impact on what can be said within such digital spaces as well as how the speech utterances and the data on the speakers are used and sold by the digital hosts. These events reiterate the comments of Butler (2015, p. 173) and others (Marquez 2012, p. 29) as to how such spaces of appearance are simultaneously a space of surveillance.

4 Macri, the Kirchners and Grindetti are Argentinian politicians and Correa is an Ecuadorean politician.

5 *Paraíso fiscal* is the Spanish translation of ‘tax haven’.

6 The regression was first run including all first-order interaction terms. For ease of interpretation, Table 3 only includes the significant interaction terms and the interaction terms for the same style component across the three speech genres. Results with all first-order interactions were similar to Table 3 in terms of sign and significance.
